# Effects of soil amendments on leaf anatomical characteristics of marigolds cultivated in cadmium-spiked soils

**DOI:** 10.1038/s41598-021-95467-9

**Published:** 2021-08-05

**Authors:** Alapha Thongchai, Weeradej Meeinkuirt, Puntaree Taeprayoon, Isma-ae Chelong

**Affiliations:** 1 Faculty of Science Technology and Agriculture, Yala Rajabhat University, Yala, 95000 Thailand; 2 Mahidol University, Nakhonsawan Campus, Nakhonsawan, 60130 Thailand; 3 Water and Soil Environmental Research Unit, Mahidol University, Nakhonsawan Campus, Nakhonsawan, 60130 Thailand

**Keywords:** Environmental sciences, Natural hazards

## Abstract

The marigolds (*Tagetes* spp.) in this study were classified as excluders for cadmium (Cd); however, their leaves also accumulated substantial Cd content. Among the experimental treatments (i.e., control, cattle manure, pig manure, and leonardite which served as soil amendments), pig manure resulted in significantly increased growth performance for all marigold cultivars as seen by relative growth rates (119–132.3%) and showed positive effects on leaf anatomy modifications, e.g., thickness of spongy and palisade mesophyll, size of vein area and diameter of xylem cells. This may be due to substantially higher essential nutrient content, e.g., total nitrogen (N) and extractable phosphorus (P), in pig manure that aided all marigold cultivars, particularly the French cultivar which exhibited the highest relative growth rate (132.3%). In the Cd-only treatment, cell disorganization was observed in vascular bundles as well as in palisade and spongy mesophyll, which may have been responsible for the lowest plant growth performance recorded in this study, particularly among the American and Honey cultivars (RGR = 73% and 77.3%, respectively).

## Introduction

Organic amendments improve soil physicochemical factors and immobilize heavy metals in soil via adsorption and isolation of cationic compounds by forming stable complexes. Soil properties affecting the availability of cadmium (Cd) include: Cd adsorption and desorption on iron (Fe) and manganese (Mn) oxides; redox potential (Eh); presence of both calcium carbonate and oxyhydroxides; pH; organic matter (OM); ionic strength; ligand anions; cation exchange capacity (CEC); phosphorus (P); and the proportion of clay minerals and percentage of charged sites occupied by Cd. Control of some of these properties can be employed to reduce Cd phytoavailability and decrease transfer of the metal into the food chain^1,2^. Furthermore, the OM content of animal manures (e.g., cattle, chicken, horse and bat) forms strong complexes with Cd resulting in the reduced mobility of Cd in soil and reduced leaching, decreasing both its toxicity and bioavailability. In one study the application of cattle manure to contaminated soil resulted in reduced Cd leaching by approximately 63%^3^. Many researchers have indicated that leonardite serves as an exceptional substitute for mineral-based fertilizer given that it is rich in humic acid and fulvic acid (50–75%). Application of leonardite (400 kg ha^−1^) and inorganics (e.g., zeolite) or organic (e.g., animal manure) materials can substantially improve the physicochemical properties of soil, leading to increased crop yield and quality^4,5^.

The leaf is the major site of photosynthesis in most vascular plants, and leaf morphology and anatomy play crucial roles in photosynthesis efficiency. Other important leaf traits such as thickness and stomatal density directly influence metal tolerance and sensitivity^6^. Physiological function, such as leaf photosynthesis and respiration, are closely associated with the changes in leaf structure, chemical function and carbon balance; the character of the changes depends on plant species and cultivar, type of root, and environmental factors^7^. Cadmium is a non-essential element for plants that can be readily taken up by root systems and transported predominantly into the aerial parts of marigolds (*Tagetes* spp.)^8^. Elevated Cd concentrations reduce plant growth by limiting rate of photosynthesis, reducing stomatal size in the lower epidermis, and reducing density, mesophyll thickness and intercellular spaces in the mesophyll^6,9^. To some extent, heavy metals i.e., zinc (Zn), mercury (Hg), lead (Pb) and Cd may cause changes in trichome length and density on leaf surfaces^10^.

Studying anatomical modifications in leaves can help in understanding biological processes in plants regarding heavy metal tolerance and accumulation mechanisms; for instance, elevated heavy metal accumulation in leaves is known to inhibit leaf transpiration rate, resulting in reduced plant growth and yields^11,12^. However, the deposition and desorption of heavy metals in leaves is known to vary by species, specific heavy metals content, speciation and certain key environmental factors, e.g., pH, cation exchange capacity (CEC) and organic matter (OM) content^13^. Comparison of leaf anatomical characteristics among various plant species grown in heavy metal-enriched soil may help identify heavy metal accumulation and patterns of distribution, and in cataloguing the effects of heavy metal toxicity and tolerance. Many plant species exposed to Cd toxicity exhibit alterations in leaf anatomical structures, examples include chickpea (*Cicer arientinum* L.) and fenugreek (*Trigonella foenum-graecum* L.)^14^. Furthermore, many reports indicate that the presence of certain essential nutrients in plant media can affect the modification of leaf anatomical characteristics. For example, Fe deficiency induces more numerous but smaller stomatal apertures in both upper and lower leaf surfaces. Similarly, large quantities of Zn have been found to cause significant reduction in epidermal cell size and area of vascular bundles, and disorganization of spongy parenchyma cells^15^.

The present study was carried out to investigate the effects of soil amendment applications on Cd accumulation in leaves, in particular the anatomical modifications in leaves of four marigold cultivars (*Tagetes erecta* L. e.g., Honey, American and Sunshine and *T*. *patula* L. e.g., French) grown in pot systems in a greenhouse environment.

## Results and discussion

### Physicochemical properties of soils

Selected physicochemical properties of soils utilized for this study are shown in Table 1. Texture of the control soil is loam, whereas the others (Cd soils) are clay. The different physicochemical properties of the experimental soils are due to both organic soil amendments and soil texture. The application of organic amendments increased soil pH and cation exchange capacity (CEC) values from 6.5 and 28.3 cmol kg^−1^ in the Cd-Alon treatment to 6.8–7.1 and 28.4–30.8 cmol kg^−1^ in the Cd-enriched soil amended with organic amendments, respectively. The desirable soil pH for optimum plant cultivation varies in the range of 5.5–7.0^16^. Soil pH is a key factor for plant growth as it can influence several physicochemical properties of soil which impact plant traits such as height, lateral spread, mass, flower size and shape, and pollen production^17^. The control soil was used in this study because it contained a very low concentration of Cd, and substantial concentrations of essential nutrients and OM that support plant growth (Table 1). The control soil amended with Osmocote^®^ showed a remarkably low CEC value; a factor of 1.6–1.7 times lower than any of the other treatments. The elevated CEC content in Cd-enriched soils led to Cd immobilization via sorption and precipitation mechanisms^18^. When comparing the differences in essential nutrients between the Cd-Alon soil and Cd soils with amendments, the organic amendments increased total nitrogen (N), extractable phosphorus (Ext. P), and extractable potassium (Ext. K) contents by approximately 1.5–2.5, 1.1–4.9, and 1–4.7 times, respectively. Highest total N and Ext. P contents were in the Cd-pig treatment (0.5% and 1483.2 mg kg^−1^). The Cd-cattle treatment also showed increased levels of Ext. P and Ext. K, at 400.2 mg kg^−1^ and 2444.3 mg kg^−1^, respectively.

**Table 1:** Physicochemical properties of the soils used in this study.

Soil properties	Treatment				
	Com-Ctrl	Cd-Alon	Cd-Pig	Cd-Catt	Cd-Leo
Soil texture	Loam	Clay	Clay	Clay	Clay
Soil pH	6.5	7.1	6.8	7.1	6.8
CEC (mol kg^−1^)	18.2	28.3	28.6	28.4	30.8
OM (%)	10.2	3.4	8.3	8.1	11.7
Total N (%)	0.4	0.2	0.5	0.4	0.5
Ext. P (%)	318.6	300.4	1483.2	400.2	322.4
Ext. K (%)	1121.4	521.4	1228.4	2444.3	5438
**Before planting**					
Total Cd (mg kg^−1^)	1.9 ± 0.2bA	8.1 ± 1.6aA	8.3 ± 0.7aA	8.3 ± 1.0aA	7.4 ± 1.0aA
**After plant harvest**					
Total Cd (mg kg^−1^)	0.9 ± 0.1bB	7.9 ± 2.4aA	7.8 ± 1.2aA	7.9 ± 1.1aA	7.0 ± 1.1abA

*CEC* cation exchange capacity, *OM* organic matter, *N* nitrogen, *Ext.* extractable, *P* phosphorus, *K* potassium, *Cd* cadmium.

Values followed by the same letter are not significantly different; lower-case letters show the difference of Cd concentrations in soils among treatments (LSD, *p* < 0.05); capital letters indicate the difference of Cd concentrations in soils before planting and after plant harvest (LSD, *p* < 0.05).

Substantial OM contents were measured in the Cd-Leo and Com-Ctrl treatments (10.2% and 11.7%, respectively), whereas lowest OM content was in the Cd-Alon treatment (3.4%). Humic substances originating from organic matter in leonardite can improve the physicochemical properties of soil and stimulate plant growth performance^19^. Rehman et al.^20^ indicated that increased OM content in amendments, applied in different combinations, could increase rice growth when high Cd-contaminated effluent water is applied to soil during planting.

Before planting, the spiked Cd resulted in significantly higher Cd contents in Cd-soils when compared with the control soil (*p* < 0.05). After plant harvest, Cd-soils showed a slight decrease in Cd content; however, there appears to be no statistically significant difference (*p* > 0.05). Several researchers have reported that organic soil amendment practices in Cd and Zn co-enriched soils were used to immobilize Cd as stable forms thus reducing mobility and bioavailability in contaminated agricultural soils, thereby markedly safeguarding human health and protecting the local environment^2^. Furthermore, Cd content decreased slightly to 2.1 times the level in the Com-Ctrl treatment (*p* < 0.05), which falls within the soil quality criteria for agricultural soil (< 1 mg kg^−1^)^21^.

### Plant growth performance

The order of dry biomass plant tissue quantities was as follows: stems > roots > leaves. Significant differences in dry biomass were recorded from cultivars grown in the Cd-pig treatment across all marigold cultivars (*p* < 0.05; Table 2). The Sunshine cultivar had higher dry biomass content in comparison to other marigold cultivars by factors of 1.5 to 47.2 times. These results are consistent with a previous study^8^. Substantial increase in dry biomass content was also measured in the Cd-cattle treatment for the Sunshine cultivar (24.6 g). The nutrient content of organic amendments such as pig and cattle manure enhance soil N concentrations considerably, which helps induce both plant growth and yield. Therefore, available N from animal manures is considered to have substantial potential and is valuable for optimum plant growth in contaminated soils. Nitrogen is also usually added as a supplement in various organic fertilizers, either alone or in combination with inorganic fertilizers^22^. The substantial OM content in the Cd-Leo treatment did not exhibit a commensurate influence on plant growth performance in this study. Although soil amendments minimize the detrimental effects of heavy metal exposure to plant cultivars, high levels of heavy metals can decrease plant biomass which might be a consequence of heavy metal stress, oxidative damage and increased membrane permeability^23,24^.

**Table 2:** Dry biomass production of the study plants (*n* = 5).

Plant	Treatment	Dry biomass production (g dw)			
		Stem	Leaf	Root	Total
French	Com-Ctrl	3.9 ± 1.1bC	0.8 ± 0.2bC	1.8 ± 1.0bB	6.4 ± 0.6bC
	Cd-Alon	1.9 ± 0.8bcB	0.1 ± 0.0cB	0.3 ± 0.2bAB	2.3 ± 0.9cB
	Cd-Pig	27.2 ± 3.2aB	2.3 ± 0.5aC	6.9 ± 4.2aA	36.3 ± 4.3aB
	Cd-Catt	3.2 ± 0.7bcB	0.1 ± 0.0cB	0.3 ± 0.1bB	3.6 ± 0.8cB
	Cd-Leo	1.9 ± 0.4cB	0.1 ± 0.0cA	0.2 ± 0.1bB	1.7 ± 0.4cC
Honey	Com-Ctrl	10.3 ± 1.3bA	1.1 ± 0.3bBC	2.4 ± 0.2bAB	13.8 ± 1.7bAB
	Cd-Alon	3.0 ± 0.7cAB	0.3 ± 0.1cA	0.4 ± 0.2cA	3.7 ± 0.9cAB
	Cd-Pig	35.3 ± 8.8aB	3.0 ± 0.5aAB	4.5 ± 2.3aA	42.7 ± 10.5aB
	Cd-Catt	1.1 ± 0.5cB	0.1 ± 0.0cB	0.2 ± 0.1cB	1.4 ± 0.5cB
	Cd-Leo	3.4 ± 0.8cA	0.1 ± 0.0cB	0.4 ± 0.1cA	3.9 ± 0.9cAB
American	Com-Ctrl	7.3 ± 1.9bB	1.2 ± 0.2bB	3.6 ± 1.3bB	12.1 ± 3.2bB
	Cd-Alon	2.1 ± 0.8cB	0.1 ± 0.0cB	0.2 ± 0.1cB	2.4 ± 0.9cB
	Cd-Pig	31.3 ± 6.7aB	2.6 ± 0.4aBC	8.1 ± 1.8aA	42.0 ± 5.9aB
	Cd-Catt	3.6 ± 2.4bcB	0.3 ± 0.1cB	0.4 ± 0.2cB	4.3 ± 2.5cB
	Cd-Leo	1.7 ± 0.9cB	0.2 ± 0.1cA	0.3 ± 0.1cA	2.2 ± 1.1cAB
Sunshine	Com-Ctrl	11.7 ± 1.0bcA	1.8 ± 0.3bA	2.2 ± 1.5bAB	15.6 ± 2.3bcA
	Cd-Alon	4.4 ± 1.9cA	0.2 ± 0.0cAB	0.4 ± 0.2bA	5.1 ± 2.1cA
	Cd-Pig	55.5 ± 17.2aA	3.5 ± 0.5aA	7.1 ± 5.8aA	66.1 ± 22.8aA
	Cd-Catt	21.3 ± 8.9bA	1.6 ± 0.5bA	1.7 ± 1.1bA	24.6 ± 9.6bA
	Cd-Leo	3.7 ± 2.0cA	0.2 ± 0.1cA	0.3 ± 0.1bA	4.2 ± 2.2cA

Values followed by the same letter are not significantly different; lower-case letters show the difference of treatments of the same cultivar (LSD, *p* < 0.05); capital letters indicate the difference in growth performance among marigold cultivars within the same treatment (LSD: *p* < 0.05).

**Figure 1:**
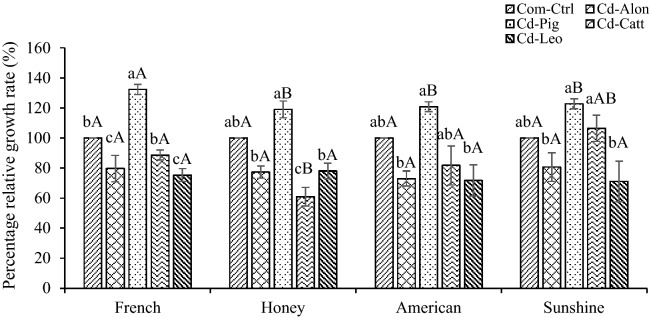
Percentage relative growth rate values of the study plants (*n* = 5). Values followed by the same letter are not significantly different; lower-case letters show the difference of treatments of the same cultivar (LSD, *p* < 0.05); capital letters indicate the difference in percentage relative growth rate values among marigold cultivars within the same treatment (LSD: *p* < 0.05).

Increased plant growth and biomass in Cd-contaminated soils treated with organic amendments were in agreement with the results of Gul et al.^25^. They reported that increased biomass of *Pelargonium hortorum* L.H. Bailey (17.9–26.6%) grown in Pb-contaminated soils was dependent upon application of compost. The descending order of dry biomass production in marigold cultivars for all treatments was as follows: Sunshine > Honey > American > French, whereas the Cd-Catt treatment showed a slightly different pattern: Sunshine > American > Honey > French (Table 2). The observed trends were slightly different for the highest % RGR values for the study plants as follows: French > Sunshine > American > Honey (Fig. 1). Furthermore, significant differences in mean values of percentage relative growth rate were observed in specimens grown in the Cd-Pig treatments for all marigold cultivars (*p* < 0.05).

### Cadmium accumulation in plant tissue

Among Cd treatments, all cultivars accumulated high quantities of Cd in whole plants, particularly in the Cd-Alon treatment for the French and Sunshine cultivars, and the Cd-Catt treatment for Honey and American treatments, ranging from 27.6 to 49.1 mg kg^−1^ (Table 3). However, marigold tissue (roots, stems, leaves and whole plant) in the Com-Ctrl treatment possessed the lowest Cd contents in all marigold cultivars (*p* < 0.05). Furthermore, all marigold cultivars accumulated lowest levels of Cd in the Cd-Pig treatment. Marigold roots accumulated the highest Cd followed by stems and then leaves, respectively, which is consistent with a previous study^8^. This plant exhibited excluder potential, that is, the propensity to accumulate Cd in lower concentrations in above-ground parts compared to roots^26^. Previous research has indicated that compost-amended soils decreased Cd bioavailability by approximately 50% in the hyperaccumulator *P*. *hortorum*, whereas compost addition reduced lead (Pb) accumulation in shoots and roots by 33% and 28%, respectively^23,27^. Furthermore, the application of organic amendments may result in restricted translocation of soluble Cd from root to shoot of the tested plants. The rather high OM content and soil pH increase Cd retention capacity, and thus decrease Cd solubility and bioavailability in soils^23,27^, as seen the remarkably low values in the treatments amended with organic amendments, particularly Cd-Pig and Cd-Leo treatments.

**Table 3:** Cd accumulation in different plant tissues from the four marigold cultivars (*n* = 5).

Marigold cultivar	Treatment	Cd accumulation in plant (mg kg^−1^)			
		Root	Stem	Leaf	Whole plant
French	Com-Ctrl	4.0 ± 1.1dA	1.7 ± 0.4cA	2.3 ± 0.6cA	2.5 ± 0.4cA
	Cd-Alon	50.4 ± 7.0aAB	37.4 ± 4.2aA	38.2 ± 3.7aA	39.0 ± 3.0aA
	Cd-Pig	28.5 ± 1.2cA	24.0 ± 2.0bA	25.6 ± 1.9bA	22.4 ± 1.0bA
	Cd-Catt	40.1 ± 6.0bAB	22.3 ± 2.7bA	25.3 ± 0.4bAB	34.0 ± 6.4abA
	Cd-Leo	39.4 ± 3.1bB	25.6 ± 4.1bA	26.9 ± 2.8bA	30.9 ± 4.1bA
Honey	Com-Ctrl	3.7 ± 1.0dA	1.4 ± 0.4cA	1.8 ± 0.7cA	2.1 ± 0.8cA
	Cd-Alon	39.7 ± 10.0abB	17.4 ± 1.7abC	23.5 ± 2.8aB	30.8 ± 3.6aB
	Cd-Pig	19.9 ± 4.0cB	10.1 ± 3.4bBC	16.6 ± 2.6bB	18.0 ± 3.3bAB
	Cd-Catt	46.4 ± 13.2aAB	20.7 ± 3.0aA	26.8 ± 0.4aAB	34.5 ± 5.2aA
	Cd-Leo	32.1 ± 3.7bC	13.8 ± 2.1bB	19.0 ± 0.8bB	22.5 ± 2.4abB
American	Com-Ctrl	3.9 ± 1.3cA	1.6 ± 0.3dA	1.9 ± 0.6dA	2.3 ± 0.6dA
	Cd-Alon	50.5 ± 4.2aA	27.5 ± 4.0abB	37.0 ± 2.8aA	41.5 ± 3.6aA
	Cd-Pig	18.8 ± 4.0bB	12.8 ± 2.6cB	16.3 ± 2.6cB	17.7 ± 3.0cB
	Cd-Catt	58.1 ± 10.2aA	29.5 ± 4.2aA	37.8 ± 6.8aA	49.1 ± 12.2aA
	Cd-Leo	52.3 ± 5.7aA	23.6 ± 6.1bA	29.5 ± 4.8bA	33.5 ± 6.2bA
Sunshine	Com-Ctrl	4.3 ± 1.3cA	1.6 ± 0.3cA	2.1 ± 0.7cA	2.9 ± 0.6cA
	Cd-Alon	48.3 ± 4.8aAB	18.6 ± 2.4aC	23.2 ± 4.4aB	27.6 ± 4.3aB
	Cd-Pig	20.5 ± 3.9bB	8.0 ± 1.9bC	9.2 ± 1.9bC	15.8 ± 3.0bB
	Cd-Catt	23.1 ± 3.8bB	10.7 ± 2.4bB	13.0 ± 1.7bB	17.3 ± 3.4bA
	Cd-Leo	19.4 ± 2.7bD	9.6 ± 2.7bB	11.7 ± 1.3bC	14.5 ± 2.7bC

Values followed by the same letter are not significantly different; lower-case letters show the difference of treatments of the same cultivar (LSD, *p* < 0.05); capital letters indicate the difference in Cd accumulation performance among marigold cultivars within the same treatment (LSD: *p* < 0.05).

### Influence of soil amendments on leaf anatomical changes

The leaves of four marigold cultivars showed slight anatomical modification in the Com-Ctrl and amended treatments (Table 4). High accumulation of Cd in leaves from the hyperaccumulating plants (e.g., marigold cultivars) varies according to plant genotype, Cd speciation and Cd quantities. Leaves can, however, potentially be seriously injured as a result of Cd toxicity^28^. Typically, elevated heavy metal content causes a decrease in the diameter and number of xylem cells, which has been reported in similar studies of certain terrestrial plant species, e.g., leaves of *Triticum aestivum* cv. Ekiz under chromium (Cr) stress^29^. Such phenomena are somewhat consistent with the present study, as leaf anatomical characteristics in the Cd-Alon treatment for some marigold cultivars showed remarkably smaller major vein areas (e.g., French, American and Sunshine cultivars) and diameter of xylem cells (American and Sunshine cultivars), compared to other treatments.

**Table 4:** Leaf anatomical characteristics among five marigold cultivars (*n* = 5).

Marigold cultivar	Treatment	Mid-vein area (µm^2^)	Adaxial-epidermis thickness (µm)	Abaxial-epidermis thickness (µm)	Mesophyll thickness (µm)	Diameter of xylem cell (µm)
French	Com-Ctrl	7731.1 ± 6488.6bB	13.7 ± 1.5cAB	17.6 ± 2.2aA	289.7 ± 79.0aA	12.7 ± 1.0bA
	Cd-Alon	3042.9 ± 2079.7bC	18.0 ± 1.5bA	23.8 ± 3.0aA	223.3 ± 66.8abA	11.3 ± 10.7bA
	Cd-Pig	24,694.5 ± 4212.1aAB	21.3 ± 2.8aA	23.0 ± 10.8aA	283.9 ± 67.4abAB	15.2 ± 2.5aA
	Cd-Catt	4723.9 ± 2496.2bB	15.7 ± 1.0bcA	16.5 ± 1.6aA	199.6 ± 14.6abBC	9.9 ± 4.1cA
	Cd-Leo	8742.1 ± 2090.1bB	14.9 ± 1.1bcA	16.8 ± 5.6aAB	182.2 ± 43.8bB	13.7 ± 3.3abA
Honey	Com-Ctrl	7191.4 ± 1333.3bB	18.3 ± 4.0aA	16.2 ± 3.1abA	219.8 ± 24.1bAB	11.4 ± 3.7abA
	Cd-Alon	7877.0 ± 3700.7bB	13.7 ± 2.0aAB	12.2 ± 2.6abC	154.4 ± 34.3bB	12.0 ± 2.5abA
	Cd-Pig	25,043.4 ± 10,954.3aAB	18.8 ± 7.3aA	17.4 ± 5.6aA	341.4 ± 83.6aA	17.1 ± 7.1aA
	Cd-Catt	12,205.9 ± 5786.5bB	13.9 ± 1.5aA	17.9 ± 6.4aA	371.5 ± 82.4aA	14.7 ± 5.8abA
	Cd-Leo	4009.3 ± 2894.0bC	7.3 ± 1.9bB	11.0 ± 2.5bB	189.4 ± 37.7bB	9.4 ± 2.2bA
American	Com-Ctrl	7713.7 ± 2121.1cB	17.7 ± 5.4aA	15.4 ± 5.7abA	151.6 ± 31.0bcB	11.0 ± 1.2bcA
	Cd-Alon	7005.2 ± 1026.8cBC	17.0 ± 4.3aA	18.6 ± 4.5aAB	166.2 ± 25.0bB	9.0 ± 3.0cB
	Cd-Pig	15,582.9 ± 5516.9aB	13.9 ± 4.8aA	19.1 ± 4.1aA	235.9 ± 24.3aB	15.8 ± 4.2aA
	Cd-Catt	12,208.9 ± 3700.8abB	13.5 ± 2.8aA	15.9 ± 2.8abA	132.9 ± 14.0cC	13.7 ± 4.1abA
	Cd-Leo	8194.7 ± 2526.4bcB	13.0 ± 2.7aA	12.8 ± 2.9bAB	173.4 ± 15.6bB	9.8 ± 3.2bcA
Sunshine	Com-Ctrl	21,444.5 ± 5608.7bA	12.1 ± 3.8bB	14.9 ± 3.4aA	225.5 ± 77.9bAB	13.6 ± 2.9abcA
	Cd-Alon	15,969.8 ± 2671.7bA	11.5 ± 3.9bB	14.1 ± 2.9aBC	262.8 ± 19.9bA	11.3 ± 2.6cA
	Cd-Pig	33,914.2 ± 7148.8aA	17.4 ± 4.9aA	17.0 ± 3.0aA	351.2 ± 97.3aA	17.4 ± 3.2aA
	Cd-Catt	35,071.8 ± 10,772.7aA	18.4 ± 2.8aA	15.2 ± 2.1aA	270.2 ± 32.6abAB	15.3 ± 3.4abA
	Cd-Leo	19,947.3 ± 3052.6bA	14.7 ± 4.3abA	18.8 ± 7.1aA	254.2 ± 46.7bA	12.6 ± 2.8bcA

Values followed by the same letter are not significantly different; lower-case letters show the difference of treatments of the same cultivar (LSD, *p* < 0.05); upper-case letters indicate the difference of leaf anatomical characteristics among marigold cultivars within the same treatment (LSD, *p* < 0.05).

Large values for major vein area and xylem cell diameter were found in plants cultivated in the Cd-Pig treatment for all marigold cultivars (15,582.9–33,914.2 μm^2^ and 15.2–17.4 μm, respectively) (*p* < 0.05), whereas almost all plants in Cd-Alon showed remarkably low values when compared to the other treatments. Plant characteristics showed significant differences in the American and Sunshine cultivars grown in the Cd-Pig and Cd-Alon treatments (2.2 and 2.1 times different, respectively). Lisa et al.^14^ found a smaller area of the vascular bundle in chickpea (*C*. *arientinum*) treated with Cd solution when compared to untreated plants. Application of organic amendments, particularly pig manure, can lead to increased mesophyll cell size and thickness of spongy parenchyma, sclerenchyma, phloem, and mesophyll in all treatments containing Cd, which can enable a greater capacity for CO_2_ retention in leaves, resulting in enhanced carbon availability and ultimately higher rates of photosynthesis^6^. To some extent, leaf mesophyll in the willow tree (*Salix viminalis* L.) stored Cd at higher concentrations when compared to veins^28^. However, in *Arabidopsis helleri* ssp. *gemmifera* Cd was localized in the vascular bundle rather than in the leaf mesophyll^30^.

**Figure 2:**
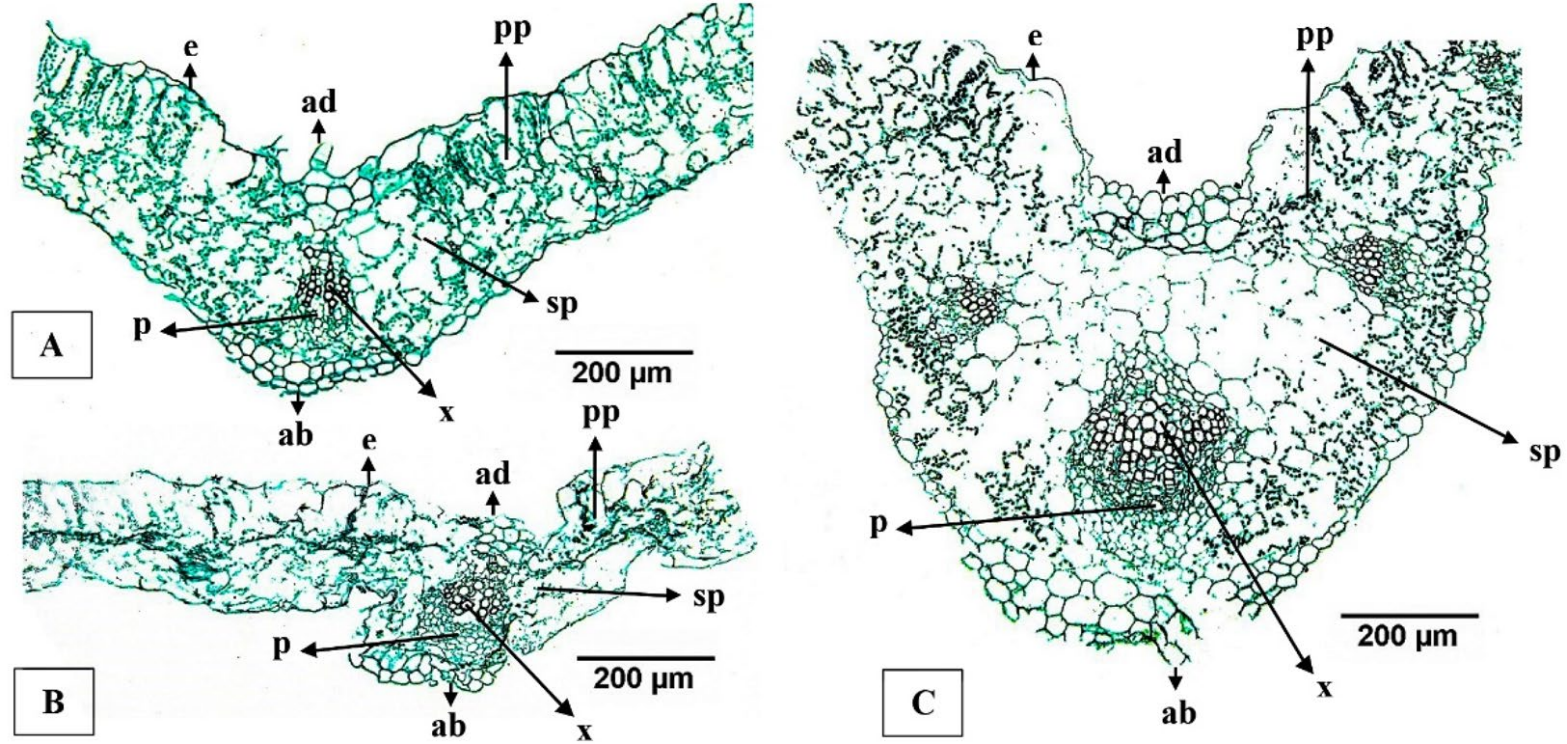
Leaf anatomy modifications occurred in Honey cultivar grown in different soil treatments (**A**) low Cd: Com-Ctrl treatment (**B**) soil without organic amendment: Cd-Alon treatment (**C**) amended soil: Cd-Pig treatment. ad = adaxial epidermis; ab = abaxial epidermis; e = epidermis; pp = palisade parenchyma; sp = spongy parenchyma; x = xylem; p = phloem.

In this study, greater mesophyll thickness was generally found in plants cultivated in the Cd-Pig treatment for all marigold cultivars (235.9–351.2 μm); however, the highest value was in the Cd-Catt treatment for Honey cultivar (371.5 μm) (*p* < 0.05). To some extent, substantial mesophyll thickness in the Cd-Pig treatment may be linked with large palisade parenchyma and spongy parenchyma cells in the mesophyll, as well as with the influence of nutrients in soil^31^, as seen in Fig. 2C for Honey cultivar and compared to the Com-Ctrl treatment (Fig. 2A). The substantial total N and extractable P contents in pig manure may be key nutrients for growth of the study plants and their development into mature plants. These nutrients may also indirectly help enhance the size and number of vacuoles in mesophyll cells, thereby alleviating Cd toxicity, as free Cd and phytochelatin (PC)-Cd complexes can be sequestered and stored in the vacuole of leaf cells^32^.

In this study, French and Sunshine cultivars grown in the Com-Ctrl treatment had significantly lower epidermis thickness on the adaxial side when compared to those grown in the amended treatments (*p* < 0.05). Similar results were observed in Sunshine cultivar grown in the Cd-Alon treatment. Furthermore, epidermis thickness on the leaf abaxial side in the Cd-Pig and Cd-Catt treatments for Honey cultivar and in the Cd-Pig treatment for American cultivar showed slight differences in comparison to the Com-Ctrl (*p* < 0.05). Some evidence indicates that heavy metals present in the growth medium can lead to increased epidermal cell size, which increases quantities of heavy metals absorbed into the epidermal layer and subsequently on the cell wall of the epidermis. This strategy helps plants prevent uptake, translocation and sequestration of heavy metals, thus reducing heavy metal absorption and/or adsorption in mesophyll cells, and increasing rates of photosynthesis^11^. The significantly elevated levels of heavy metals on the epidermal cell walls, however, can cause deformation and breakdown of chloroplasts and many cell organelles, including cell walls of mesophyll^33^. Furthermore, an elevated soil Cd content could substantially reduce the photosynthetic rate in the mesophyll of the hybrid *Pennisetum* grass. However, the toxicity threshold for Cd in plants varies among plant species and within a species among subspecies, ecotypes, cultivars and even tissues^9^.

Small major vein areas and diameters of xylem cells were typical of all marigold cultivars grown in the Cd-Alon treatment; these characteristics were clearly visible in the study plants grown in the Cd-Pig treatment across all marigold cultivars. This finding was in contrast with a previous study, as Cd accumulation in vascular bundle of the major vein did not affect xylem vessel diameter in *Typha domingensis* Pers.^34^. A measure of disorganization and shrinkage of epidermal cells and disorganization of mesophyll and vascular bundle material were observed in Honey cultivar grown in the Cd-Alon treatment (Fig. 2B); although similar observations were made in the American cultivar, they were more modest with slight disorganization in vascular bundle and a small amount of shrinkage in epidermal cells. Such phenomena have caused severe decline of cell viability and a reduction in thickness and breakdown of the palisade and spongy mesophyll layer, mainly as a result of Zn or Cd stress^35^. Among cultivars grown in amended treatments, only the French cultivar in the Cd-Catt treatment developed small major vein areas, phloem degradation, a small degree of shrinkage in epidermal cells and slight disorganization of mesophyll. To some extent, a disorganized and degraded mesophyll layer caused by Cd exposure can eventually interfere with photosynthetic efficiency, photosystems and other multiprotein complexes (MPCs) in thylakoids, resulting in decreased plant growth and productivity^36^.

Venation patterns of the study plants were observed with a light microscope. The marigold cultivars had slightly different densities and numbers of minor veins; specimens of the Sunshine cultivar grown in the Cd-Leo treatment exhibited the highest number. Cultivars were ranked in order of decreasing minor vein density as follows: Sunshine > American > Honey > French; ranking as a function of treatments is: Cd-Leo > Cont > Cd-Catt > Cd-Pig > Cd. Minor veins play a key role in heavy metal mobility, and contaminants primarily assimilate in the leaf mesophyll at the mature stage. High quantities of Cd can be detected in major veins within the leaf mesophyll and mesophyll tissues during the development period, whereas very low Cd accumulation was found primarily in vascular bundles^37,38^. However, the rate of water and solute (including heavy metal) uptake and transport from veins across the mesophyll to the point of evaporation from aerial parts, depends on plant genotype, hydraulic capacity, and photosynthetic mechanism^39^.

Notably, specialized structures of the epidermis, e.g., trichomes, were found in Honey cultivar grown in Cd-Pig and Cd-Leo treatments as were the American cultivar grown in the Cd-Alon treatment; no such structures developed in any specimens of the French and Sunshine cultivars regardless of treatment type. Increased heavy metal stress can result in enlargement of trichomes, whose structures and size can act as barriers preventing heavy metal adsorption and accumulation via leaf surfaces. However, there is still no clear understanding of the mechanism by which trichomes detoxify heavy metals from leaf surfaces. Ricachenevsky et al.^40^ hypothesized their utility regarding metal detoxification in plant leaves, as *Arabidopsis thaliana* (L.) Heynh. accumulated substantial concentrations of Zn in trichomes when compared to other plant organs, which may be considered as a key mechanism for alleviating metal toxicity on plant grown in contaminated soils. The presence of trichomes, and their structure and number on leaf surfaces probably depends on several physicochemical and biological factors, including the presence of heavy metals^41,42^.

## Conclusions

We conclude that organic amendments, particularly pig manure, could reduce the deleterious effects of Cd toxicity and support growth performance of the study plants, as indicated by leaf anatomical changes. The obvious results i.e., cell disorganization in vascular bundles and palisade and spongy mesophyll, were found in the Cd-Alon treatment which contained high quantities of Cd and no organic amendments.

## Methods

### Plant materials

Seeds of four marigold cultivars, including *T*. *erecta *e.g., Honey, American and Sunshine, and *T*. *patula *e.g., French, were purchased from local agricultural shops in Nakhonsawan Province, Thailand. Seeds were sterilized in 10% sodium hypochlorite (NaOCl) for 1 min and then germinated in acid-washed sand with total and extractable Cd levels below detectable limits. All seeds were transferred to a greenhouse at the Nakhonsawan Campus, Mahidol University. Conditions in the greenhouse were temperatures within 27–32 °C, ~ 65% relative humidity and ~ 16,000 lx.

### Greenhouse experiments

All experiments were carried out according to ethical principles on plant usage. Prior to planting, all soil samples were oven-dried and passed through a 2-mm sieve. Then, each organic amendment (10% w/w) i.e., pig and cattle manure and leonardite, was mixed thoroughly with Cd-enriched soil collected from Mae Sot, Tak Province. Treatments were designated as Cd-Pig, Cd-Catt, and Cd-Leo; a Cd-enriched soil with no amendment was designated as Cd-Alon. Furthermore, commercial soil amended with Osmocote^®^ (slow-release inorganic fertilizer; 0.15%) also served as control soil; designated as Com-Ctrl. The control soil was the mixture of fermented sugarcane bagasse, sugarcane filter cake, soil and dolomite; thus, soil was friable and suitable for plant cultivation. Cd-enriched soils were spiked with Cd(NO_3_)_2_ to attain measured values of 8.1 ± 1.6, 8.3 ± 0.7, 8.3 ± 1.0 and 7.4 ± 1.0 mg Cd kg^−1^ for Cd-Alon, Cd-Pig, Cd-Catt and Cd-Leo treatments, respectively, whereas the control soil, purchased from an agricultural supplies shop, contained low concentrations of Cd (1.9 ± 0.2 mg Cd kg^−1^). All pots were saturated with deionized water (DI water) for 4 weeks at 70% water holding capacity. Selected physicochemical properties of the soil samples were analyzed following standard methods e.g., texture, pH, CEC, OM, total nitrogen (total N), extractable phosphorus (Ext. P) and extractable potassium (Ext. K)^43,44^.

Marigold seedlings (~ 10 cm height at 7 days) were transferred to 3.5-kg size plastic pots and placed on greenhouse benches in a randomized complete block design (RCBD). Five replicate pots were used for each treatment, and each replicate comprised a single healthy seedling. The pot treatment systems were designed and modified by the methods of Thongchai et al.^8^.

### Cadmium determination

At 3 months after planting, plants were harvested and oven-dried at 70 °C for 3 days. For each sample, shoots, roots, leaves and whole plant tissues were ground to a fine powder with an IKA^®^ A11 basic mill. Plant materials were digested with concentrated 70% HNO_3_ and 37% HCl (1:3)^43^. Cd contents in solution were analyzed using flame atomic absorption spectrophotometry (FAAS; Perkin Elmer AAnalyst 200, USA) after filtering with Whatmann^®^ No. 42 filter paper.

Two standard reference materials (NIST SRM^®^ 2710a Montana soil and NIST SRM^®^ 1515 apple leaves) for soil and plant samples were used for the verification of the accuracy of analytical measurements. Percentage recoveries of Cd for the soil and plant materials were in the range of 90–110% of the stated content, whereas the relative standard deviation (RSD) ranged from 1.14 to 4.01%.

### Light microscopy

At plant maturity the middle portions of the leaves were cut into small pieces (0.5 × 0.5 cm^2^) and immediately fixed in FAA (formaldehyde: glacial acetic acid: 95% ETOH: distilled water; 5:5:50:40 mL) for 48 h as described by Johansen^45^. The specimens were gradually dehydrated using a graded series of tertiary butyl alcohol (TBA) and then embedded in paraffin wax. Sections were cut at 5 μm with a motorized rotary microtome (ERM 4000, HESTON). The sections were subsequently counterstained with fast green and safranin^45^. After staining, slides were dehydrated, cleaned with fresh xylene and mounted with Permount^®^ for examination and photographing structural changes of leaf cells under a Leica DM1000 LED microscope with a Leica MC170 HD camera. Measurement of the lumen diameter of xylem cell, mesophyll thickness, major vein area, and epidermal cell size on both adaxial and abaxial sides of the leaf were carried out using the ImageJ program (version 1.52v).

### Data analyses

Dry weight of shoots, roots, leaves and whole plant tissues were recorded. Furthermore, relative growth rate (RGR) index data were calculated as described by Phusantisampan et al.^46^, affording a metric of plant growth tolerance in extreme environmental conditions.

Statistical analysis was carried out using SPSS^®^ (SPSS, Chicago, IL) on a Windows-based PC. A one-way ANOVA and least significant difference (LSD) post hoc comparison were used to detect significant differences in the mean values (*p* < 0.05) and are presented as mean ± standard deviation.
